# How to Prevent Iodine from Staining

**Published:** 1869-02

**Authors:** 


How to Prevent Iodine from Staining.—The addition of carbolic acid to the tincture of Iodine not only imparts antiseptic properties, but renders the iodine colorless. Dr. Boulton, of London, recommends the following prescription in chronic ozœna, to be applied by the spray producer; compound tincture of iodine, 1 drachm; solution of carbolic acid (as sold in the shops), 6 gtt.; water, 6 oz.

